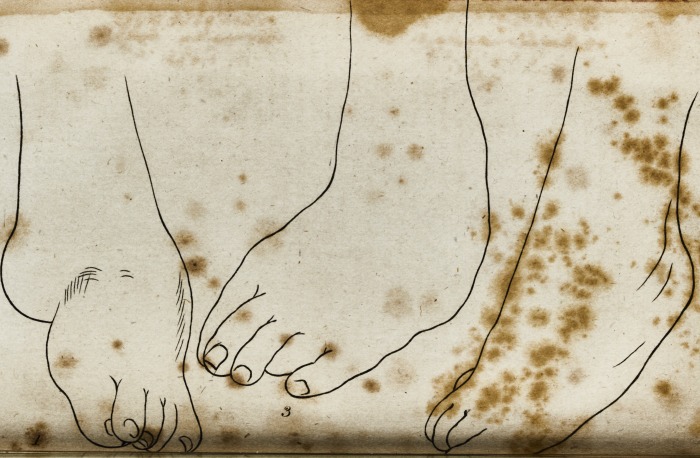# Mr. Sheldrake, on the Club-Foot

**Published:** 1800-12

**Authors:** T. Sheldrake

**Affiliations:** No. 50, Strand


GENTLEMEN,

I Shall now, with your permiſſion, reſume the ſubject I hinted at in my laſt Communication, viz. to ſhow, as far as my experience will ſhow, under what circumſtances, and at what periods of life, thoſe diſtortions of the feet, which are commonly called Club-feet, and more learnedly, but more vaguely, termed varus and valgus, may be cured. Two methods of doing this may be adopted; the firſt, to advance the doctrine we mean to ſupport, and as far as we can, demonſtrate the truth of it by facts to be afterwards produced: the ſecond, to relate the facts, and afterwards draw thoſe conſequences that ſeem to be fairly deducible from them.

The firſt mode of proceeding has the appearance of advancing an hypotheſis, and to mere hypothetical reaſoning upon practical ſubjects, many, with juſtice, will object; and when a prejudice is thus excited in the mind, it will, ſometimes, not be obliterated by the ſubſequent production of facts; but when facts are ſimply demonſtrated, they are received without prejudice, and every unprejudiced reader will clearly ſee, whether the conſequences drawn from them regularly follow, or whether they admit of a different interpretation. As the laſt mentioned mode of proceeding appears to me to be the beſt, I ſhall adopt it, and ſelect from my notes a few caſes, which appear to me to be remarkable, and which certainly would not be generally credited, without the ſtrong proofs I ſhall produce to confirm them; I ſhall then draw ſuch concluſions from them as they will fairly admit of, and which, I am not without hopes, may amount to a demonſtration of the nature of the diſeaſe in general, and the poſſibility of curing it under circumſtances in which it has not till now been cured, though the cure has ſometimes been attempted: The firſt of theſe caſes I ſend with this, and will ſend the remainder as ſoon as my other avocations will afford me time to prepare them. I am,

GENTLEMEN,

Your moſt obedient ſervant,

T. SHELDRAKE.

*No.* 50, *Strand*,

*Nov.* 5, 1800.

---

### CASE I.

In the beginning of January, 1798, Mr. Pugh, who then reſided in Percy Street, requeſted me to ſee a patient at his

 houſe; houfe; he was a youth of 11 years old, a native of Mofcow, and had been born with the left foot diftorted; every thing that the ftate of fcience, in his own country, could fuggeft, had been tried in vain; he then came to London, and was confided to the care of Mr. Pugh, who defired a confultation with Meffrs. Cruikfhank and Wilfon; Mr. Pugh propofed to have the tibial mufcles and aponeurofis plantaris, or fuch parts of them *as he thought* were contracted, divided; after which, he faid, *he imagined* he could compleat the cure; this was objected to by the two gentlemen, and Mr. Wilfon advifed that I fhould be confulted.

Dr. Grieve, Mr. Pugh, and the patient's uncle, were prefent when I examined the foot. I had reafon to believe it was a cafe that I could cure; but I felt it would be indelicate to propofe, that the patient fhould be taken from Mr. Pugh and confided to me, I therefore only faid, I thought he might be much benefited by what I could do for him; and it was agreed that I fhould do what I thought right, Mr. Pugh at the fame time continuing to treat him in his way. But, in a private conference with Mr. Pugh, after the patient and his friends had retired, I defired to know *his opinion* of the cafe? He thought the patient might be benefited, but had no conception that a cure was poffible; I declared, unequivocally, my conviction that he might *be perfectly cured*; but, I added, it muft be by means only known to myfelf, and by my own application; that I had not mentioned this, from motives of delicacy, before the patient and his friends, but thought it right to inform him, I fhould do nothing unlefs the patient was left entirely to my management; and if this was not agreeable to him, I fhould decline interfering, and confider myfelf as withdrawn from the cafe.

Mr. P. was delighted to hear that I thought a cure was not impracticable, and laboured ardently to perfuade me to *inform him* of the method I fhould ufe, and put *him* in poffeffion of my inftruments; but finding me inflexible, he, with *the affectation of much liberality*, *declared* he only fought for the patient's benefit, and therefore confented that I fhould proceed in my own way.

But ere I was prepared to begin, the uncle called upon me; he apologized for the difagreeable ftep he was going to take, but added, that Pugh had defired him to prevent *me* from doing any thing, as fome ideas had occurred to *him*, which he hoped would be beneficial to the patient, and he accordingly did fatisfy me for my trouble, and the cafe was again left to Mr. Pugh.

In the beginning of February, however, the fame gentleman applied to me again; I learned, that during the time that elapfed elapfed fince I laft faw him, Mr. Pugh had only repeated his former practice, but he had introduced fome workman who made an inftrument that could not be applied, he therefore was now determined to truft the cafe entirely to my management.

At this time I had a caft in plafter of Paris made from the foot; this caft remains in my poffeffion, and will demonftrate that the facts were as follow:

The foot was perfectly rigid, and he was incapable of moving it voluntarily in any direction; though, when he ftood upon it, the little toe only touched the ground, and then the ancle joint bent a little under him, yielding to the weight of the body, and, in confequence of this action, becoming gradually more deformed: the head of the aftragalus projected much before the tibia, the tarfal bones formed an acute angle with refpect to the tibia, and bent inwards: the metatarfal bones likewife formed an acute angle with refpect to the bones of the tarfus; there was no voluntary motion in the toes, and the tibial mufcles and plantaris aponeurofis *feemed* to be permanently contracted. The knee, in confequence of the imperfect action of the foot, had bent confiderably inwards.

He continued under my care till June 19, in the fame year, when it was neceffary that he fhould return to Ruffia; at this time I had a fecond caft taken from the foot, which, as well as the former, remains in my poffeffion, and will demonftrate the following facts, viz.

He ftood perfectly flat on his foot, inftead of bearing only on the little toe, the top of the foot forming a right angle with the leg as much as any other foot does: the head of the aftragalus had regained its natural pofition under the fcaphoid cavity of the tibia; the bones of the tarfus had regained their natural pofition relative to the leg, and the metatarfal bones had almoft got into their natural pofition: the confequence of all this was, that the foot had acquired a form nearly refembling the natural foot, and actually meafured feven-eights of an inch *more in length than it did when I firft faw it, four months before this period.* The plantaris aponeurofis and tibial mufcles had loft their apparent rigidity, and, as well as the tendo achillis, or rather the gaftrocnemii mufcles, which move it, had acquired the power of acting in the natural way; the peronei mufcles, as well as thofe which move the toes, had acquired their natural action, and he was, in confequence, enabled to move the foot in every direction, and in perfect obedience to the will.

So much having been done in this cafe, there was every reafon to believe the cure would have been perfect in every fenfe, had he continued longer under my care; but it was indifpenfibly difpenfibly neceffary that he fhould return to Ruffia; and it was hoped that what little was ftill wanting to compleat the cure, might be affected by means of the inftruments with which I fupplied him. His tutor, an intelligent man, who paid particular attention to the inftructions I gave him, undertook the charge; I have fince enquired of Mr. N. the patient's uncle, who is ftill in London, and from him have received the following letter.

Monsieur, *Oct$^r$.* 15, 1800.

Ayant lû la defcription que vous avez fait de l'etat dans lequel le pied de mon neveu fe trouvoit avant qu'il fut remis entre vos mains et de la difference tres marqûee, que le fuccés des foins et des moyens que vous avez employés y avoit déja porté; lorfqu'a mon grand regret, forcé par quelques circonftances particulieres, je me vis obligé de le renvoyer dans fa patrie, et de faire cefer, ainfi, fon traitement. Je croirois, Monfieur, totallement manquer aux fentimens de juftice, et a ceux de la reconnoiffance pour le bien que vos foins ont rendus a un jeune homme qui m'intereffe, fi je ne faififfois pas, avec empreffement l'occafion que vous m'offrez de fatisfaire au defir que j'ai toujours eu, de rendre a vos talents un temoignage auffi public que poffible; guidé par ce fentiment, ainfi que par l'amour de la juftice, je declare que tout ce que j'ai lû dans la memoire que vous m'avez fait l'honneur de me communiquer, porte, en general, et fur tous fes points, le caractere de la plus ftricte verité; et que, de mon coté je n'ai pas le moindre doute que s'il eut été poffible de le laiffer plus long tems entre vos mains, fa jambe rentreroit entierement dans fon etat naturel. En effet, Monfieur, je crois que l'evidence des faits m'autorife de le fuppofer: car, lofqu' aprés m'avoir feparé de lui pour quelques mois, je le revis à Hambourg, par ou il paffoit pour rentrer dans fon pais, je ne fus pas peu furpris de le voir pofer fa jambe fur la plante du pied, et d'une maniere qu'il ne refteroit rien a defirer une fois que le vice du genou, fucceffivement produit par le poids du corps, qui, dans fon etat, devoit neceffairement porter a faux, feroit enlevé: et, en examinant la derniere machine que vous fites pour lui et dont il devoit continuer a s'en fervir, j'ai crû avoir toute les raifons poffibles de former les plus flateufes efperances que ce derniere moyen remedieroit à ce mal, avec le meme fuccés qu'ont eu les premiers. Je fuis faché, pourtant, que malgré toutes mes inftances pour avoir quelques details fur la fuite des effets qu' a produit l'application de cette derniere machine, je ne puis obtenir d'autres reponfes, que celles, ou l'on me difoit vaguement, que fa jambe alloit beaucoup mieux. Je le repête repête encore que, pour ma propre ſatisfaction ainſi que pour la votre, j'aurois bien deſiré pouvoir etre en etat de vous en rendre un compte plus circonſtantiè; mais, en attendant que je ſois a meme de le faire, je ne ſuis cependant pas peu flaté de pouvoir vous rendre toute la juſtice qui eſt en mon pouvoir, comme un tribût de la reconnoiſſance avec laquelle j'ai l'honneur d'etre,

Monsieur,

*No. 71, Warren Street, Fitzroy Square.*

Votre trés devoué,

N. de Novassilzoff.

It will, perhaps, not be unreaſonable to conclude, that ere this the patient is *perfectly* well; but as I wiſh to avoid every thing that may afford the leaſt pretence for doubt or contradiction, I ſhall confine myſelf to ſuch facts as are capable of being ſatisfactorily demonſtrated.

I have ſaid I cauſed one caſt to be made from the foot before any thing was done, and another at the time I took my leave of the patient; theſe caſts are in my poſſeſſion, and I have made three drawings from each of them, placing them as nearly as poſſible in correſponding ſituations; theſe views are annexed, and will convey an accurate idea of the ſtate of the foot at the periods when it was within the ſphere of my obſervation. Mr. N's letter deſcribes what was his ſituation ſome months after he left me; and from the combined effect of this evidence, the reader will form his own concluſions.

In making my drawings of the original diſeaſe, I have placed the leg quite upright, and the foot falls into the ſituation it was in when he ſtood upon it: There are three views of the foot in this ſtate; No. 1, looking directly upon the inſide of the leg; No. 2, upon the outſide of the ſame; and No. 3, a front view of the leg. The appearance of the foot in each of theſe views, will, I think, ſhow very intelligibly the actual ſtate of the diſtortion.

In drawing the foot after it was cured, I have purſued the ſame plan; that is, there are three views; in making which, I placed the leg in exactly the ſame ſituation as I did the former; by comparing them, the improved ſtate of the foot will, I think, be clearly underſtood.

In No. 1, being the inſide of the foot, there appears not the leaſt deformity; in No. 2, the appearance is not ſo perfect; and in No. 3, there is the additional diſadvantage of ſhowing a fore ſhortened view of the foot: but theſe ſhow all the deformity then remaining, viz. that thoſe bones which form what is commonly called the inſtep, are ſomething higher than uſual.

At

At the time this patient was entrufted to my care, I had treated none who were by many years fo far advanced in life. The circumftance which then feemed to me remarkable, as it diftinguifhed this from every cafe that had been under my care, was the following: In young fubjects, the reduction of the feet to their natural form is effected long before the power of ufing them is acquired; but in this cafe, thofe two effects were produced at the fame time; whence we are juftified in concluding, that if thofe trifling peculiarities which remained when he was taken from under my care, were not afterwards removed, notwithftanding thofe imperfections, his foot is in every refpect as ufeful as the other; but if we are to affume, as moft probably is the fact, that they have fince difappeared, the cure is in every refpect compleat.

---

### *To the Editors of the Medical and Phyfical Journal.*

Gentlemen,

## Figures and Tables

**F.1. F.2. F.3. F.4. f1:**
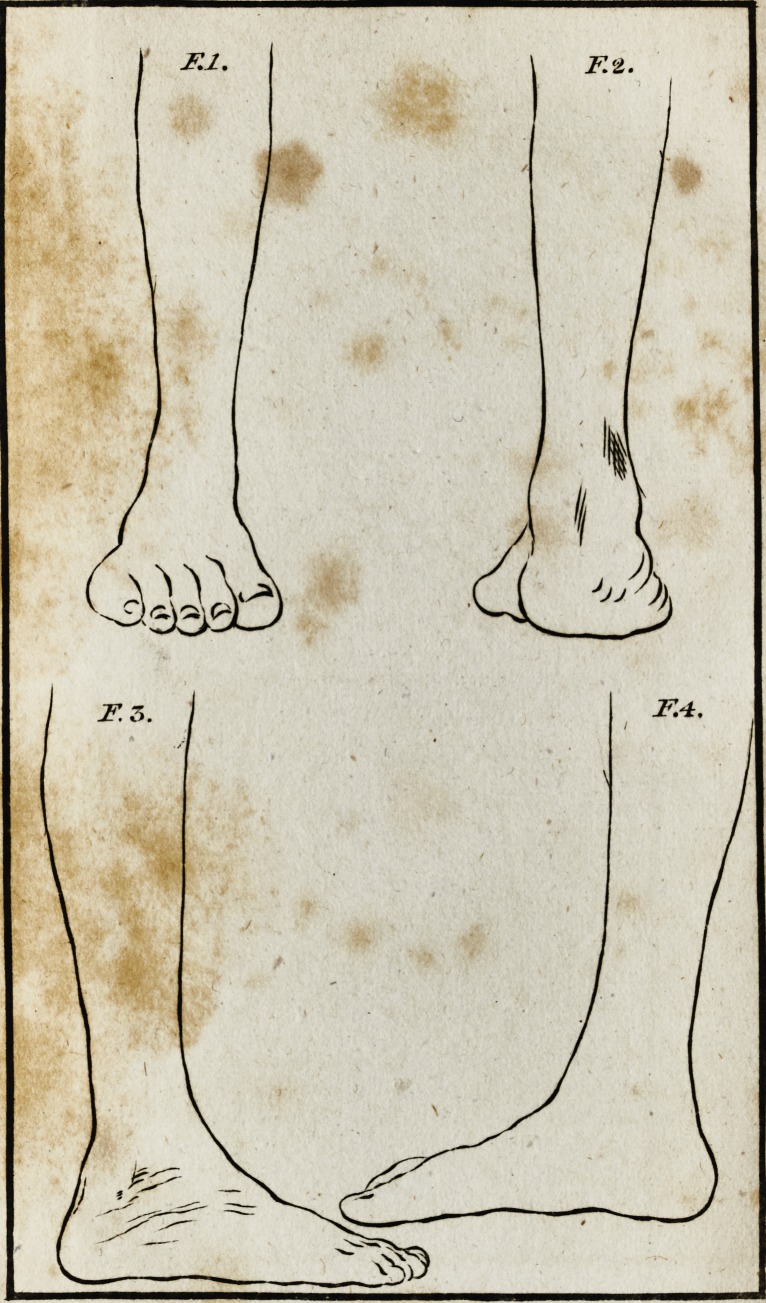


**F.1. F.2. F.3. F.4. f2:**
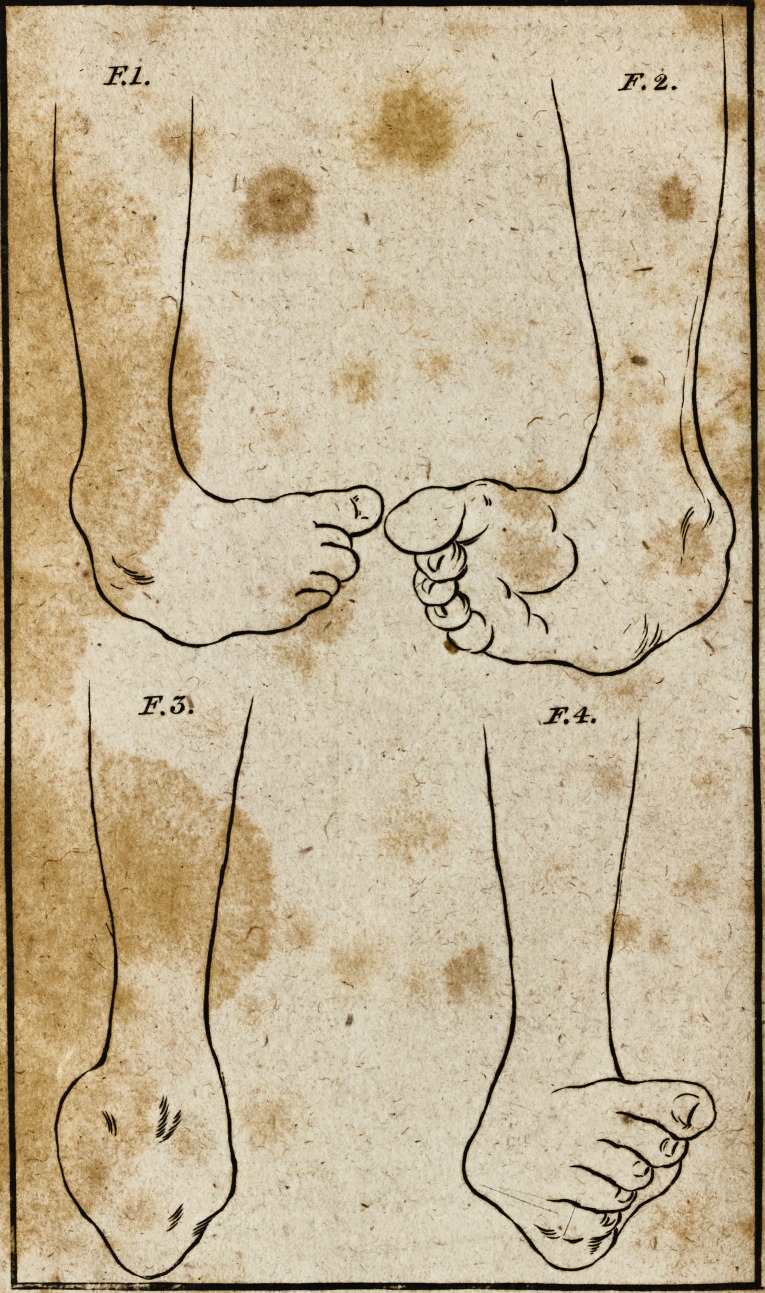


**1 2 3 f3:**
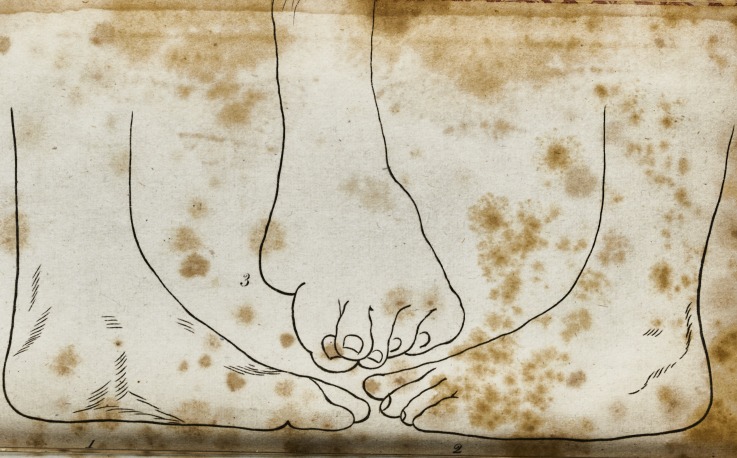


**1 3 f4:**